# Cytokine-Induced Killer Cells Express CD39, CD38, CD203a, CD73 Ectoenzymes and P1 Adenosinergic Receptors

**DOI:** 10.3389/fphar.2018.00196

**Published:** 2018-04-20

**Authors:** Alberto L. Horenstein, Antonella Chillemi, Roberta Zini, Valeria Quarona, Nicoletta Bianchi, Rossella Manfredini, Roberto Gambari, Fabio Malavasi, Davide Ferrari

**Affiliations:** ^1^Laboratory of Immunogenetics and CeRMS, Department of Medical Sciences, University of Torino, Torino, Italy; ^2^Centre for Regenerative Medicine “Stefano Ferrari,” Department of Life Sciences, University of Modena and Reggio Emilia, Modena, Italy; ^3^Department of Life Science and Biotechnology, Section of Microbiology and Applied Pathology, University of Ferrara, Ferrara, Italy

**Keywords:** CIK, adenosine, CD38, CD39, CD73, CD203a/PC-1, hypoxia, P1 receptors

## Abstract

Cytokine-induced killer (CIK) cells, a heterogeneous T cell population obtained by *in vitro* differentiation of peripheral blood mononuclear cells (PBMC), represent a promising immunological approach in cancer. Numerous studies have explored the role of CD38, CD39, CD203a/PC-1, and CD73 in generating extracellular adenosine (ADO) and thus in shaping the tumor niche in favor of proliferation. The findings shown here reveal that CIK cells are able to produce extracellular ADO via traditional (CD39/CD73) and/or alternative (CD38/CD203a/CD73 or CD203a/CD73) pathways. Transcriptome analysis showed the mRNA expression of these molecules and their modulation during PBMC to CIK differentiation. When PBMC from normal subjects or cancer bearing patients were differentiated into CIK cells under normoxic conditions, CD38 and CD39 were greatly up-regulated while the number of CD203a, and CD73 positive cells underwent minor changes. Since hypoxic conditions are often found in tumors, we asked whether CD39, CD38, CD203a, and CD73 expressed by CIK cells were modulated by hypoxia. PBMC isolated from cancer patients and differentiated into CIK cells in hypoxic conditions did not show relevant changes in CD38, CD39, CD73, CD203a, and CD26. CIK cells also expressed A_1_, A_2A_, and A_2B_ ADO receptors and they only underwent minor changes as a consequence of hypoxia. The present study sheds light on a previously unknown functional aspect of CIK cells, opening the possibility of pharmacologically modulated ADO-generating ectoezymes to improve CIK cells performance.

## Introduction

CIK cells are polyclonal T effector cells sharing immunological properties and receptors with NK cells. They are attracting increasing interest for their ability to perform non-MHC-restricted cytolytic activities toward susceptible autologous and allogeneic cancer cells (Schmidt-Wolf et al., 1991; Lu and Negrin, 1994). This capacity, along with their safety and easy *in vitro* generation has opened the door to multiple applications of CIK adoptive immunotherapy against different types of cancer. Hence, CIK can be employed against solid and hematological tumors, either alone or together with chemotherapy. An experimental CIK based approach has been undertaken for the following neoplasms: chronic and acute lymphocytic leukemias, lymphomas, kidney carcinoma, renal, liver and stomach cancer, melanomas and bone sarcomas (Linn and Hui, 2010; Gammaitoni et al., 2013; Jiang et al., 2013; Sangiolo et al., 2014). CIK cells are generated by *ex vivo* cultivation of human PBMC in the presence of the cytokine interferon gamma (IFN-ɤ), the anti-CD3 monoclonal antibody OKT3, and then adding recombinant human IL-2 (rhIL-2) (Introna et al., 2007; Jiang et al., 2013; Giraudo et al., 2015). The addition of IFN-γ has the main goal of activating monocytes present in the mixed PBMC population to secrete IL-12, which favors CD58/LFA-3-mediated activation, while the binding of anti-CD3 antibody to CD3 membrane antigen expressed by T lymphocytes and the addition of IL-2 provides cells with the mitogenic stimuli they need for proliferation (Franceschetti et al., 2009). CIK cells are a heterogeneous population comprising CD3^+^CD8^+^ T cells, CD3^+^CD56^−^ T cells (from 20 to 60% of total CIK), and CD3^+^CD56^+^ double positive cells (from 40 to 80% of total CIK), as well as of a small number of CD3^−^CD56^+^ NK cells (from 1 to 10%) (Franceschetti et al., 2009; Pievani et al., 2011; Introna et al., 2013; Valgardsdottir et al., 2014).

Immune cells interact with cancer cells in the so called “tumor niche,” i.e., in a localized neoplastic tissue context; therefore they are heavily influenced by the superimposed tumor conditions. Some of the most influential extracellular mediators in the niche are the nucleotides and nucleosides. Adenosine (ADO), the main nucleoside mediator generated both intracellularly and extracellularly, suppresses the anti-tumoral immune response, thus favoring metastasis to the detriment of the host organism. Once present in the extracellular *milieu*, nucleotides and nucleosides bind purinergic receptors, i.e., specific plasma membrane receptors necessary for cell-to-cell communication and named P2 (activated by ATP, UTP, ADP, UDP, UDP-glucose, and NAD^+^) and P1 receptors (G protein-coupled, activated by ADO). P1 are further divided into four subtypes (A_1_R, A_2A_R, A_2B_R, A_3_R) (Burnstock, 2007; Surprenant and North, 2009; Harden et al., 2010; Plattner and Verkhratsky, 2016).

Extracellular nucleotides and nucleosides are subjected to continuous transformation. The main canonical actors of this function are four ectonucleotidases: ectonucleoside triphosphate diphosphohydrolase (NTPDase, CD39), ectonucleotide pyrophosphatase/phosphodiesterase (NPP, CD203a) or PC-1, ecto-5′nucleotidase (CD73), and alkaline phosphatases (Yegutkin, 2008; Zimmermann et al., 2012) which degrade ATP and its metabolites, eventually leading to ADO production and the subsequent activation of P1 receptors (Fredholm et al., 2001). Transformation of extracellular ATP into its metabolites requires the sequential participation of the CD39 ectoenzyme (stepwise forming extracellular AMP) subsequently metabolized by CD73 into ADO (Yegutkin, 2008; Zimmermann et al., 2012).

Another pathway generating extracellular AMP, which can then be transformed into ADO, involves participation of extracellular NAD^+^ and CD38 an ADP-ribosyl cyclase/cyclic ADP-ribose (cADPR) hydrolase. Expression of CD203a (ectonucleotide pyrophosphatase/phosphodiesterase-1) accompanied by the extracellular presence of its substrate ADPR (product of the deconstruction of NAD^+^ by CD38) favors the generation of additional AMP (Horenstein et al., 2013).

ADO plays multiple roles as an extracellular mediator both in physiological and pathological conditions. It is released in different tissue contexts, including neurons, kidney cells, cardiomyocytes, vascular endothelium and immune cells (Shryock and Belardinelli, 1997; Latini and Pedata, 2001; Dale and Frenguelli, 2009; Praetorius and Leipziger, 2010; Morandi et al., 2015; Silva, 2016). However, the interest in ADO and ectoenzymes involved in ADO formation (CD38, CD39, CD73 and CD203a) was prompted by growing evidence of their role in cancer biology. Indeed, ADO suppresses immune responses against tumor cells, and cancer-derived ADO is recognized as a crucial extracellular immune checkpoint target for re-establishing immune-surveillance mechanisms (Young et al., 2014; Hatfield and Sitkovsky, 2016; Allard et al., 2017).

A role in favoring cancer growth and dissemination has been described for each of the extracellular enzymes involved in nucleotide/nucleoside and NAD^+^ metabolism (i.e., CD39, CD38, CD203a and CD73) and for the complexing ADO deaminase (ADA)/CD26 molecules, a controller of ADO in the extracellular space. CD39 is involved in colorectal cancer dissemination (Stagg and Smyth, 2010) as well as in the metastatic competence of non-small-cell lung cancer (NSCLC) (Schmid et al., 2015; Ferrari et al., 2017). CD73 is involved in the spread of cancer and in reducing immune-surveillance (Wang et al., 2008; Antonioli et al., 2016a,b; Ferrari et al., 2017). This is achieved by using antibodies to CD73 and CD39 (Young et al., 2014; Allard et al., 2017; Kazemi et al., 2017) to block ADO, a normal immune regulator, which is hijacked by tumors to evade immune attack. Likewise, human CD38 (Malavasi et al., 2008) is implicated in multiple myeloma, where it is main target of therapeutic treatments (Horenstein et al., 2015; Sanchez et al., 2016; Costa et al., 2017; Shallis et al., 2017). Concerning P1, A_2A_R and A_2B_R have been indicated as the main candidate receptors in cancer therapy (Beavis et al., 2013; Desmet et al., 2013; Hatfield and Sitkovsky, 2016; Allard et al., 2017; Garber, 2017; Mittal et al., 2017).

However, so far, there is no information on P1, CD39, CD38, CD203a, CD73, and CD26 expression in CIK cells. In view of the important immunosuppressive role played by ADO and the promising use of CIK cells in adoptive cancer therapy, we investigated the metabolic processes of ADO generated during the differentiation of human T cells present into the PBMC fraction into CIK cells under normoxic and hypoxic conditions.

## Materials and methods

### CIK cell production and characterization

PBMC cells from 11 donors, five healthy donors, five patients with histologic confirmed Gastrointestinal Stromal Tumors (GIST) and 1 patient with osteosarcoma (OS). Blood samples were obtained through an ongoing collaboration with Dr D. Sangiolo at the Candiolo Cancer Institute FPO-IRCCS Fondazione del Piemonte per l'Oncologia. Patients provided written informed consent for blood donation according to a protocol approved by the internal review board and ethic committee (Ethic Committee, IRCCS Candiolo Cancer Institute, Turin, Italy. Prot. CE IRCCS 244/2015).

Cryopreserved PBMC were seeded at a concentration of 2x10^6^ cells/ml according to the standard protocols (Gammaitoni et al., 2013; Sangiolo et al., 2014), including 21 days of culture in RPMI-1640 medium (Gibco BRL Life Technologies Italia, Monza, Italy) supplemented with 10% fetal bovine serum (Sigma Aldrich, MI, Italy) 100 U/ml penicillin, and 100 U/ml streptomycin (Gibco BRL Life Technologies Italia, Italy) at 37°C and 5% CO_2_, with the timed addition of IFN-γ (1,000 U/ml on day 0), Ab anti-CD3 OKT3 (50 ng/ml on day 1) and IL-2 (300 U/ml on day 1 up to the end, refreshing the medium every 2-3 days) (all factors are from Miltenyi Biotec S.r.l., Calderara di Reno, BO, Italy). In parallel with the standard *ex vivo* cultures, at day 0 an aliquot (7 × 10^6^) of PBMC was seeded (2 × 10^6^ cells/ml) in RPMI-1640 medium with 10% fetal bovine serum, 100 U/ml penicillin and streptomycin at 37°C and 5% CO_2_) but without the addition of INF-γ to perform mRNA analysis. At day 1 of culture, 3 × 10^6^ of these cells were collected for RNA extraction. Cells were lysed in Invitrogen™ TRIzol™ (Thermo Fisher Scientific S.p.a., MI, Italy) and stored at−80°C. RNA extraction was repeated with the same procedure for each CIK cells cultures at day 14 and 21.

Phenotype of CIK cells was weekly analyzed starting from day 0 by standard flow cytometric assays. The following monoclonal antibodies (mAb) were used: CD3-FITC, CD4-PE, CD56-APC, CD8-PE, and CD314-APC (anti-NKG2D) (all mAb are from Miltenyi Biotec S.r.l., BO, Italy). Labeled cells were read on FACS Cyan (Cyan ADP, Beckman Coulter s.r.l., Cassina De' Pecchi, MI, Italy) and analyzed using Summit Software.

### Evaluation of ectoenzyme expression on CIK cells by flow cytometry

FACS analysis of CD56^+^CD3^+^ CIK cells was performed using FITC-labeled anti-CD56 (Beckman Coulter Inc., Brea CA, USA) and PE-Cy7-labeled anti-CD3 antibodies (BioLegend, Milan, Italy). Expression of ectoezymes was detected by using the following mAbs generated and purified in-house by two-step HPLC chromatography (Horenstein et al., 2003) and APC-conjugated by Aczon (BO, Italy): anti-CD38 (clone IB4), anti-CD73 (clone CB73), anti-CD203a (clone 3E8, kindly provided by J. Goding) and anti-CD26 (clone BT5.9). CD39 expression was analyzed using anti-CD39 APC mAb (clone eBioA1, eBiosciences, San Diego, CA, USA). Tests were performed on cells washed in phosphate buffered saline (PBS) containing 1% bovine serum albumin (BSA) + NaN_3_ and incubated with APC-conjugated mAb for 1 h at 4°C. The samples were then washed, resuspended in PBS and acquired on a FACSort flow cytometer (Becton-Dickinson, USA) using CellQuest Software (Becton-Dickinson). Data were analyzed using FlowJo Software (TreeStar).

Expression of ADO receptors was evaluated on CIK cells gated for CD3^+^ CD56^+^ and assayed in PBMC and in the corresponding CIK cells using the following antibodies: purified rabbit polyclonal anti-A_1_R (LifeSpan BioSciences, Inc., USA), rabbit polyclonal anti-A_2A_R and rabbit polyclonal anti-A_2B_R (Thermo Scientific, USA). PE-conjugated goat anti-rabbit Ig (Beckman Coulter, USA) was used as secondary reagent. Data were expressed as mean relative of fluorescence intensity (MRFI), obtained as follows: mean fluorescence obtained with specific mAb/mean fluorescence obtained with irrelevant isotype-matched mAb.

For FACS analysis under hypoxic culture conditions, total cryopreserved PBMC were seeded at a concentration of 2 × 10^6^ cells/ml in a humidified CO_2_ incubator (Thermo Scientific Water Jacketed 3010) and differentiated into CIK cells by using the standard procedure. Culture conditions were 21% (normoxia) or 1% (hypoxia) O_2_, 5% CO_2_ at 37°C. The phenotype was weekly analyzed starting from day 0 by standard flow cytometric assays.

### Quantification of ADO production by CIK cells

CIK (1 × 10^6^/ml) were incubated in 500 μl HBSS at 37°C and 5% CO_2_ in 24 well plates (Costar Corning) and pre-treated with 50 μL of stop solution [EHNA (5 μM) + Dipyridamole (20 μM) + Levamisole (30 μM) + Deoxycoformicin (50 μM)], in the presence (or absence) of NAD^+^, ADPR, ATP or AMP (100 μM). At the end of the incubation time (30 min), supernatants were collected and acetonitrile (ACN, Sigma Aldrich) was immediately added (1:2 ratio) to stabilize ADO. Samples were then centrifuged at 13,700 × g, collected and stored at −80°C until use. The presence of NAD^+^, ATP, ADPR, AMP, and ADO was investigated by high-pressure liquid chromatography (HPLC, Beckman Gold 126/166NM, Beckman Coulter) equipped with a reversed-phase column (Synergi Polar C18, 5 μm; 150 × 4.5 mm, Phenomenex). The metabolites were separated using a pH 5.1 mobile-phase buffer (0.125 M citric acid and 0.025 M KH_2_PO_4_ containing 8% ACN) over ten min with a flow rate of 0.8 mL/min and UV detection set at 254 nm. Peak identities were confirmed by using standard compounds. The presence of ADO was also confirmed by spiking standard (50 μM ADO), followed by chromatography. The retention times (Rt, in min) of standards were: ATP, 2.00; AMP, 2.35; NAD^+^, 2.8; ADPR, 3.44, and ADO; 5.56. All concentrations measured on CIK-derived supernatants were normalized to cell number and volume.

### Expression profile of adenosinergic ectoenzymes and P1 purinergic receptors in CIK cells

The mRNA expression data of PBMC and CIK cells originate from Mesiano et al. (2017) and were deposited in the Gene Expression Omnibus repository (https://www.ncbi.nlm.nih.gov/geo/query/acc.cgi?acc=GSE97581).

Briefly, we performed the gene expression profile (GEP) of PBMC (day 1, absence of INF- γ) and CIK cells (day 14) obtained from 3 GIST patients by means of HG-U219 Array Strip (Affymetrix; Santa Clara, CA, USA) (Mesiano et al., 2017). Microarray data were analyzed by using the Partek GS 6.6 Software Package and normalized using the robust multiarray average (RMA) procedure (Irizarry et al., 2003).

Differentially expressed genes (DEGs) were then selected using a supervised approach with the ANOVA module included in Partek GS package. In particular, we considered differentially expressed genes (DEGs) all the probe sets with a fold change contrast ≥ 1.4 in the pairwise comparison of CIK cells with PBMCs, and a false discovery rate (FDR) (*q*-value) < 0.5.

### Statistical analysis

*p*-value was calculated using an unpaired nonparametric test, two-tailed Mann-Whitney for GraphPad Prism 6. Reported data are expressed as mean values ± SD.

## Results

### Transcriptome analysis on adenosinergic ectoenzymes

**Figure 1:**
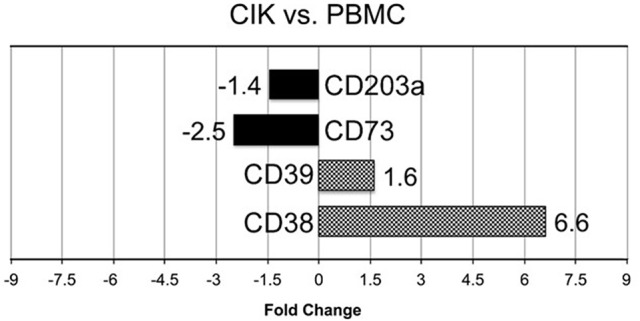
Transcriptome analysis of CIK ectoenzymes. mRNA expression was analyzed in PBMC (day 1, absence of INF-γ) and CIK cells (day 14) obtained from 3 GIST patients. The black blocks display the transcripts that are down-regulated during CIK differentiation; whereas, the chess pattern displays the mRNAs that are up-regulated during CIK differentiation.

*In vitro* differentiation of CIK cells starts with cultivation of PBMC according to standard protocols (Gammaitoni et al., 2013; Sangiolo et al., 2014). To shed light on extracellular enzymes involved in nucleotide/nucleoside and NAD^+^ metabolism we checked the expression levels of CD39, CD38, CD203a and CD73 during CIK cell differentiation. As shown in Figure 1, CD38 and CD39 were up-regulated in CIK cells form 3 GIST subjects (day 14) vs. PBMC (day 1, absence of INF-γ), whereas CD73 and CD203a resulted down-regulated.

### Expression of nucleotide-hydrolyzing ectoenzymes in PBMC and CIK cells

**Figure 2:**
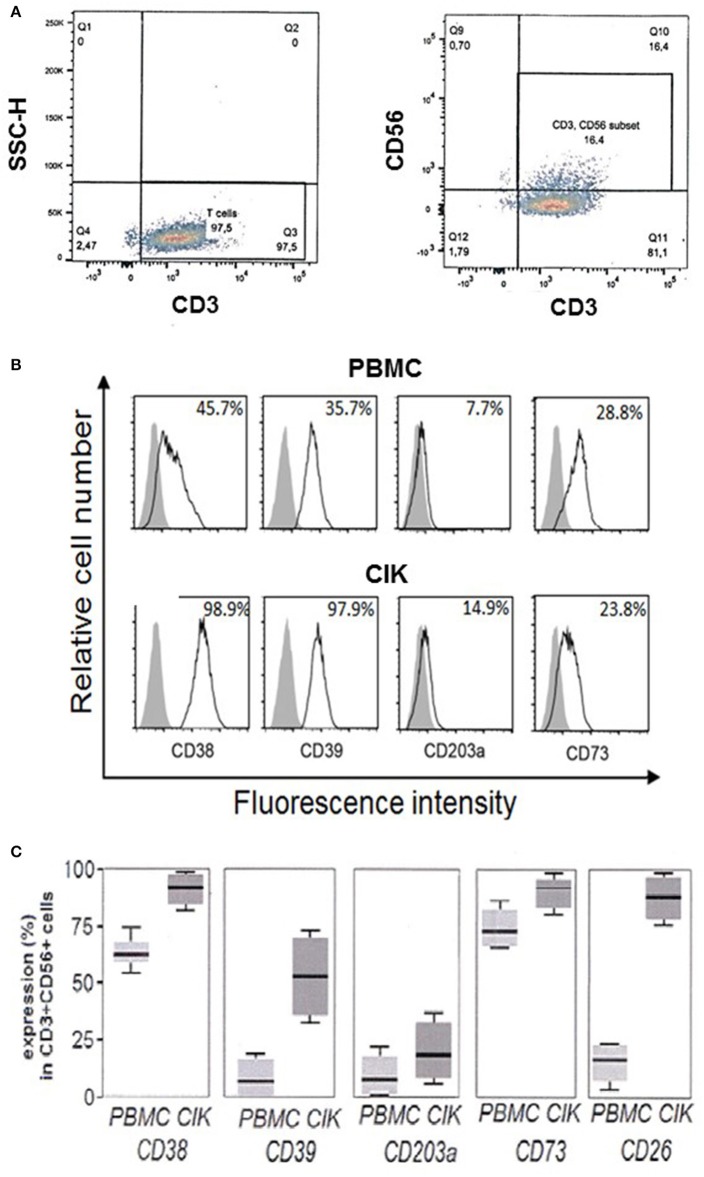
Expression of CD38, CD39, CD203a, and CD73 in CD3^+^CD56^+^ human PBMC and in the corresponding CIK cells. **(A)** Representative gating of CD3^+^ and CD3^+^CD56^+^ populations present in CIK cells at the third week of culture. Analysis was performed by using the fluorophores PE-Cy7 or FITC for CD3 and CD56 antigens, respectively. **(B)** Cytofluorometric expression analysis of ectoenzymes (white peaks) in PBMC at day 0 (upper panel) and CIK cells at day 21 (lower panel). CD38, CD39, CD73, and CD203a were detected by APC conjugated antibodies. Gray peaks demarcate isotype control staining. Percentage of positive cells is indicated. **(C)** Percentage of expression of the different markers in PBMC (day 0) and CIK cells (day 21). Cells were from 6 subjects (5 gastrointestinal stromal tumors and 1 osteosarcoma). In **C**, data are expressed as mean, minimum and maximum values.

Firstly, cell cultures were gated for CD3^+^CD56^+^ expression and CD38, CD39, CD203a, and CD73 were assayed both in PBMC and in the corresponding CIK cells. A representative gating of the CIK population at the third week of culture is shown in Figure 2A. Expression of the assayed markers is represented by white peaks (Figure 2B). Cytofluorometric analysis revealed that the NAD^+^-consuming CD38 ectoenzyme was present in 45.7% of the PBMC while it was expressed by the vast majority (98.9 ± 1%) of CIK cells, with minor variations in mean fluorescence intensity (MFI; mean ± SD, 148 ± 25). The expression of CD39 increased during PBMC to CIK differentiation (PBMC 35.7 ± 1.89; CIK 97.9 ± 2.34). CD203a and CD73 were also monitored (Figure 2B). In contrast to CD38 and CD39, CD203a was barely expressed by PBMC and increased in CIK cells (PBMC 7.7 ± 0.29; CIK 14.9 ± 0.34); while expression of CD73 was almost unchanged during differentiation (28.8 ± 3.65 vs. 23.8 ± 12.1).

While day 0 corresponds to withdrawal of PBMC, day 21 is usually chosen as end of *in vitro* differentiation period to start procedures for CIK cell infusion into cancer patients. Figure 2C shows that CD3^+^ CD56^+^ CIK cells from 6 subjects 5 GIST and 1 OS) maintained the initial high levels of CD38 with a mean value of 62.5% (min 53.2%, max 75.0%) vs. 92.5% (min 81.5%, max 98.8%); CD39 had a mean value of 13.2% (min 2.3%, max 23.5%) vs. 53.3% (min 32.2%, max 75.2%) associated to a significant increase at day 21 of the expression of CD26 as compared to resting PBMC with a mean value of 20.5% (min 6.8%, max 23.1%) vs. 91.2% (min 76.5%, max 96.5%), along with minimal CD203a up-regulation with a mean value of 15.1% (min 2.5%, max 24.1%) vs. 23.5% (min. 13.6%, max 35.1%), and stable expression of CD73 with a mean value of 75.2% (min 63.5%, max 80.30%) vs. 93.6% (min 77.5%, max 97.6%). These results indicate that CIK cells are provided with a complete ectoenzymatic machinery able to produce ADO through the traditional (CD39/CD73) as well as alternative (CD38/CD203a/CD73 or CD203a/CD73) pathways.

### Extracellular ADO production by CIK cells

The role of ADO as potent suppressor of immune-surveillance has been well ascertained and has adopted by many cancer types as a mechanism of escape from the deleterious activity of macrophages, dendritic cells, T lymphocytes and NK (Morandi et al., 2015; Antonioli et al., 2016a,b). For adoptive immunotherapy CIK cells are usually infused as bulk into tumor bearing patients. Therefore, we checked whether the adenosinergic ectoenzymes expressed by bulk CIK cells deriving from PBMC of cancer patients were functional. To address this issue, we evaluated ADO produced by CIK cells obtained from 6 subjects (5 GIST and 1 OS). ADO release was investigated in supernatants from CIK cells incubated with NAD^+^ (CD38 substrate), ADPR (CD203a substrate) and AMP (CD73 substrate) in the presence of EHNA (adenosine deaminase inhibitor).

#### Metabolism of extracellular NAD^+^

**Figure 3:**
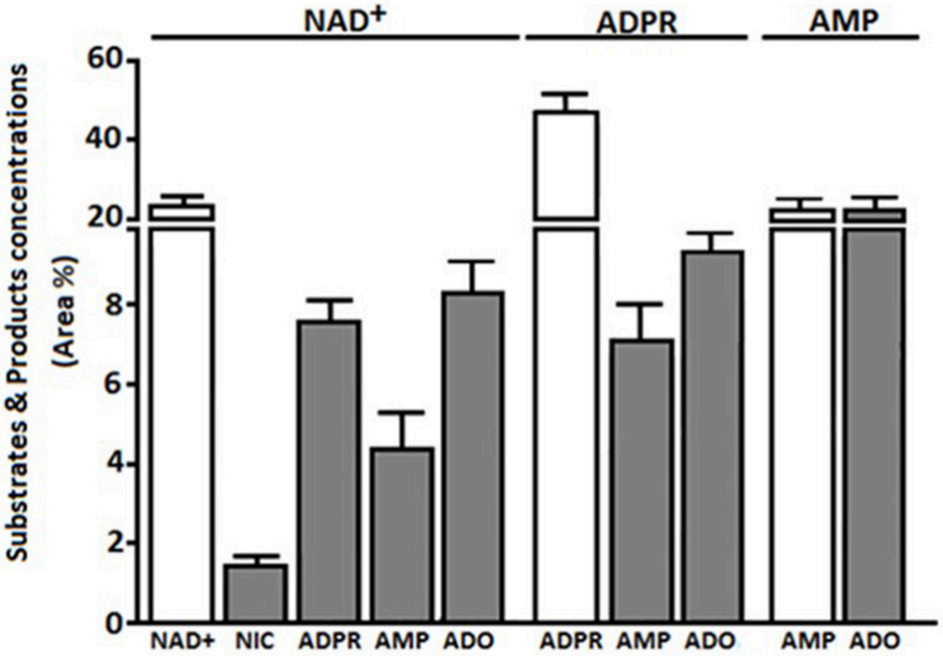
Extracellular products of CIK enzymatic reactions using NAD^+^, ADPR, and AMP as substrates. Products (NIC, ADPR, AMP, or ADO) obtained from bulk CIK cell cultures (*n* = 5) using (i) NAD^+^, (ii) ADPR or (iii) AMP as substrates, were evaluated by HPLC assays in the presence of the adenosine deaminase inhibitor (EHNA) as described in Materials and Methods. Products (gray bars) are expressed as area percentage (Area %) for each enzymatic product as compared to the total components present in the ACN-treated CIK cell supernatant (100%). Substrates are represented as Area % of consumed substrate (white bars). Results indicate that bulk CIK cells efficiently hydrolyze NAD^+^, ADPR and AMP to generate ADO. Of note, CIK cells produce low or undetectable amounts of ADO when incubated with NAD^+^, ADPR, or AMP substrates in the absence of EHNA (not shown). The identity of peaks was confirmed by the co-migration of reference standards.

HPLC-assisted analysis of the supernatants of bulk CIK cells revealed the presence of non-consumed NAD^+^ together with the enzymatic products ADPR and nicotinamide (NIC) (*n* = 5) (Figure 3). Besides the non-consumed NAD^+^, which could be identified in the HPLC assay by its unique retention time (R_t_) of 2.80 min, the metabolic products of CIK cells were ADPR, NIC and AMP, exhibiting corresponding R_t_ of 3.44, 6.87, and 2.35 min, respectively.

The NAD^+^ hydrolytic profile of CIK cells, converting extracellular NAD^+^ into AMP, can be attributed to the ADP-ribosyl cyclase/cyclic ADPR-hydrolase activity of CD38 and CD203a, which exhibits nucleotide pyrophosphatase/phosphodiesterase activity. ADPR produced by CD38 upon the partial breakdown of NAD^+^ can subsequently be degraded to AMP by CD203a, confirming the observations reported in other cell systems (Horenstein et al., 2015, 2016).

Since ADPR was the principal product of exogenously applied NAD^+^, we wondered whether it would be source of the AMP generated by CIK cells. Thus, we tested the functional activity of CD203a by directly applying ADPR, a known substrate for this ectoenzyme (Figure 3). CIK cells displayed an ADPR-hydrolyzing activity leading to AMP, supporting our hypothesis that the NAD^+^ converting pathway leading to AMP is operative in CIK cells concomitantly expressing CD38 and CD203a.

#### Metabolism of extracellular ATP

**Figure 4:**
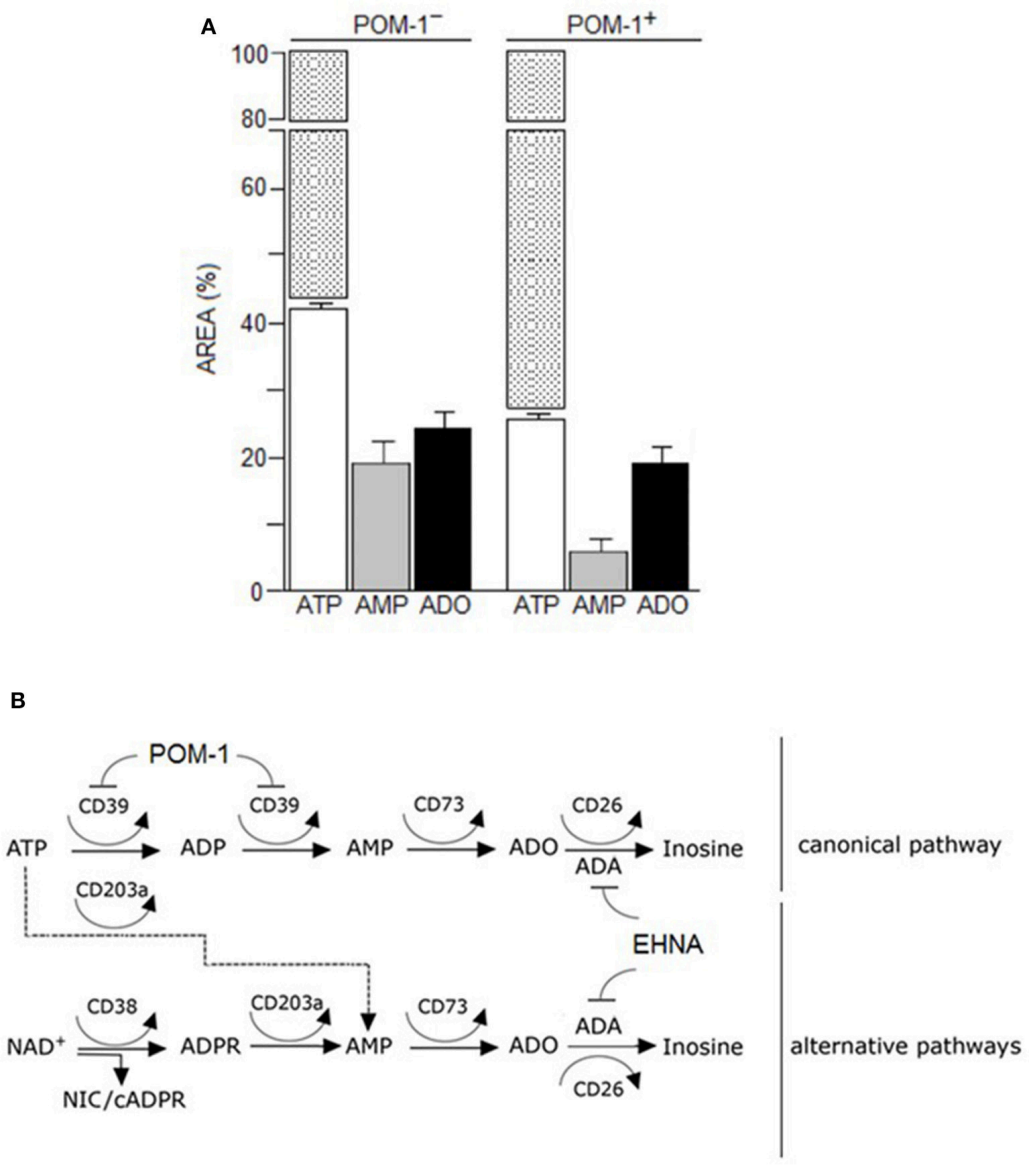
Canonical adenosinergic pathway in CIK cells. **(A)** To prove the existence of ATP adenosinergic pathway in CIK cells, production of ADO was investigated in CIK supernatants collected 30 min after 100 μM ATP addition. White columns indicate the concentration of consumed ATP in the presence or absence of POM-1 (inhibitor of CD39). White/dotted columns indicate the total area of the substrate ATP (100%). Generated products (AMP and ADO) are indicated by grey and black columns, respectively. Data are expressed as area percentage (AREA %) of AMP and ADO. **(B)** Canonical and alternative scheme pathways of ADO production showing the enzymatic targets of POM-1 (inhibitor of CD39) and EHNA (inhibitor of adenosine deaminase, ADA).

The canonical adenosinergic pathway in CIK cells was investigated using ATP, a known substrate for CD39 or alternatively for CD203a (Figures 4A,B). Therefore, analysis (*n* = 5) of ATP consumption by CIK cells allowed a comparison of both nucleotide transformation pathways. The incubation of CIK cells with ATP led to a predominant accumulation of AMP, with low levels of ADP due to the catabolism of ATP by CD39 ectoenzyme (Figure 4A). However, at variance with reports pointing to the exclusive CD39-mediated ATP hydrolysis to ADP and AMP by lymphoid cells, CIK cells converted part of the ATP substrate directly to AMP, confirming the simultaneous presence of functional CD203a. This finding was confirmed by the attenuation (~50%) of the metabolic conversion of ATP into AMP in the presence of the CD39 inhibitor POM-1 (Figures 4A,B). These experiments support the view that the enzymatic activity of CD203a produced AMP also by a secondary conversion of ADPR (arising from the breakdown of NAD^+^ by CD38), in a CD39-independent manner.

#### Metabolism of extracellular AMP

The 5′-NT CD73 is expressed on the surface of select lymphoid cells. Thus, the heterogeneous T cell population composing CIK cells, ought to display AMP-degrading activity toward ADO generation. Indeed, these cells provided the proper phenotypic background with the capacity to degrade extracellular AMP to ADO (see the complete cellular phenotype of CIK cells in Figure 1).

HPLC experiments confirmed that CIK cells dephosphorylate extracellular AMP (Figure 3). HPLC analysis showed that AMP was dephosphorylated to ADO: AMP (R_t_ = 2.35 min) was metabolized (~80% within 30 min) by CIK cells resulting in a production of ADO (R_t_ = 5.56 min). Low level of inosine (INO, R_t_ = 3.26 min) was detected in the absence of EHNA (CD26 inhibitor) (not shown). Inhibition experiments further evinced the role of CD73 in the conversion of AMP. When CIK cells were incubated with AMP in the presence of α,β-methylene-ADP (APCP, a CD73 inhibitor), the catabolism of AMP and the formation of ADO were strongly decreased (~80%). These results indicate that the CD73 was the predominant ectoenzyme participating in ADO generation by CIK cells.

#### Metabolism of extracellular ADO

Extracellular ADO may partially accumulate in the culture medium (or tumor extracellular *milieu*) where it binds specific P1 receptors or be internalized through nucleoside transporters. Alternatively, surface adenosine deaminase (ADA), complexed to CD26, converts ADO to INO (Figure 4B). PBMC expressed CD26 (6.8%) and during the cytokine-dependent differentiation to CIK cells the molecule raised to 89.4%. We confirmed such feature of CIK cells by measuring the increment of ADO production upon the incubation of these cells with AMP in the presence of EHNA (an inhibitor of ADA). Consequently, ADA on the surface of CIK cells, anchored to the CD26 receptor, offer to a combination of ectoenzymes [CD38 (cyclase/hydrolase), CD203a (NPP) or CD39 (NTDase) along with CD73 (5′-NT)] a machinery for ADO generation (Figure 4B). Indeed, results obtained indicated that consumption of 50% of the added ATP produced AMP (20%) and ADO (23%) after 30 min of incubation (Figure 4A). The presence of ADO indicated that the ectoenzyme clustering on the surface of CIK cells leaded to the production of the nucleoside. The consumption of NAD^+^ by CIK cell in 30 min was, however, kinetically slower. As shown in Figure 3, consumed NAD^+^ (22%) leaded to the production of 8% of ADPR, 5% of AMP and only 8% of ADO. Taking into consideration that low metabolization of AMP as substrate (20%) was paralleled by a high efficiency of ADO production (20%) confirmed the observation of a reduced expression of CD203a. Alternatively, a plausible explanation is a low enzymatic efficiency of CD203a to catalyze the conversion of ADPR into AMP. Accordingly, ADO production by CIK cells using NAD^+^ was lower than using ATP either by CD39 or CD203a. In conclusion, CIK cells are equipped with a functional ectoenzymatic machinery leading to ADO production in the extracellular *milieu* (~25 μmol/min/10^6^ cells). However, CIK cells produced low amounts of ADO when incubated with NAD^+^, ADPR, AMP, or ATP as substrates in the absence of EHNA (CD26/ADA tandem inhibitor), confirming the presence of ADA (evaluated by measuring the expression of the surrogate CD26 molecule) as shown in Figure 3.

### Expression of P1 receptors during CIK cell differentiation

**Figure 5:**
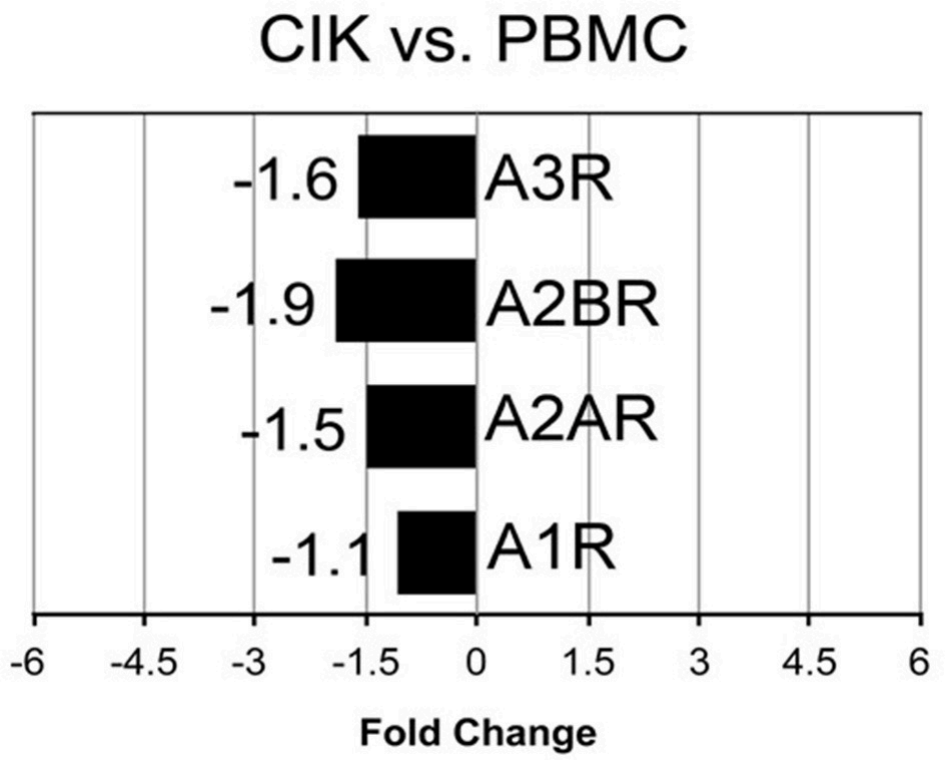
Transcriptome analysis of P1 receptors in CIK cells. mRNA expression of A_1_R, A_2A_R, A_2B_R, A_3_R was analyzed in PBMC (day 1, absence of INF-γ) and CIK cells (day 14) obtained from 3 GIST patients as described in Materials and Methods.

Cellular responses to ADO are induced through activation of four subtypes of specific G protein-coupled receptors (A_1_R, A_2A_R, A_2B_R, and A_3_R) (Chen et al., 2013). Extracellular ADO generated by canonical (CD39/CD73) or alternative (CD38/CD203a/CD73 and CD203a/CD73) pathways, can be captured by the cells, thereby explaining its local signaling. Therefore, next step was to detect at mRNA and protein levels, the expression of these receptors during CIK cell differentiation. As shown in Figure 5, mRNA expression level of A_2A_R, A_2B_R, and A_3_R were decreased in CIK cells compared to PBMC, while A_1_R expression was not changed (*n* = 3).

To further investigate CIK immune regulation mechanisms, we analyzed the expression of ADO receptors at the protein level. Results obtained in additional patients confirmed that CIK cells of further expressed A_1_R, A_2A_R, and A_2B_R (see below), a feature that can be exploited by CIK cells to modulate intracellular cyclic AMP (cAMP). A_2A_R plays a role in lymphocyte deactivation by ADO and accumulation of high extracellular ADO in the absence of ADA is lymphotoxic (Huang et al., 1997; Burnstock and Boeynaems, 2014). Hence, to support cytotoxic CIK properties, the final product of the adenosinergic reaction might be devoid of an autocrine cAMP-dependent signaling. To comply with this condition, we hypothesized that a CIK active ADA/CD26 complex might scavenge pericellular ADO from the extracellular environment to facilitate their own survival and to protect its cytotoxic activities. In fact, we were unable to detect physiological levels of ADO when HPLC assays were carried in the absence of EHNA, an inhibitor of adenosine deaminase (Figure 3).

### CIK cell ectoenzymes and purinergic receptors in hypoxic conditions

**Figure 6:**
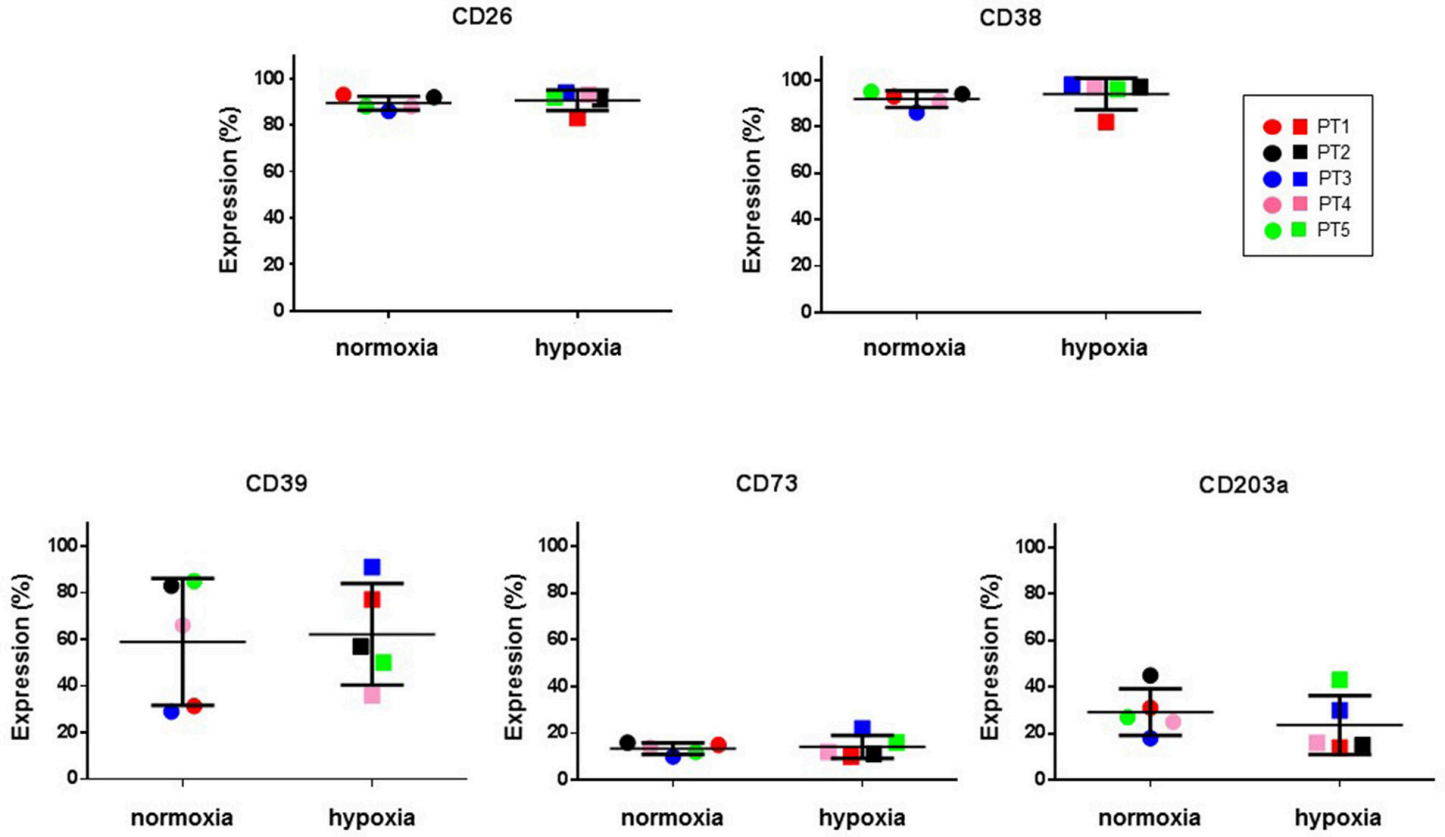
Expression of ectoenzymes in CIK cells under normoxic and hypoxic conditions. Comparative cytofluorimetric analysis of CIK cells stained with primary antibodies to various ectonucleotidases as reported in Materials and Methods. To compare hypoxia and normoxia we used unpaired nonparametric test, two-tailed Mann-Whitney for GraphPad Prism 6. Circles, normoxia; squares, hypoxia. Patients are as following: PT1, red; PT2, black; PT3, blue; PT4, pink; PT5, green. Reported data are expressed as mean values ± SD.

**Figure 7:**
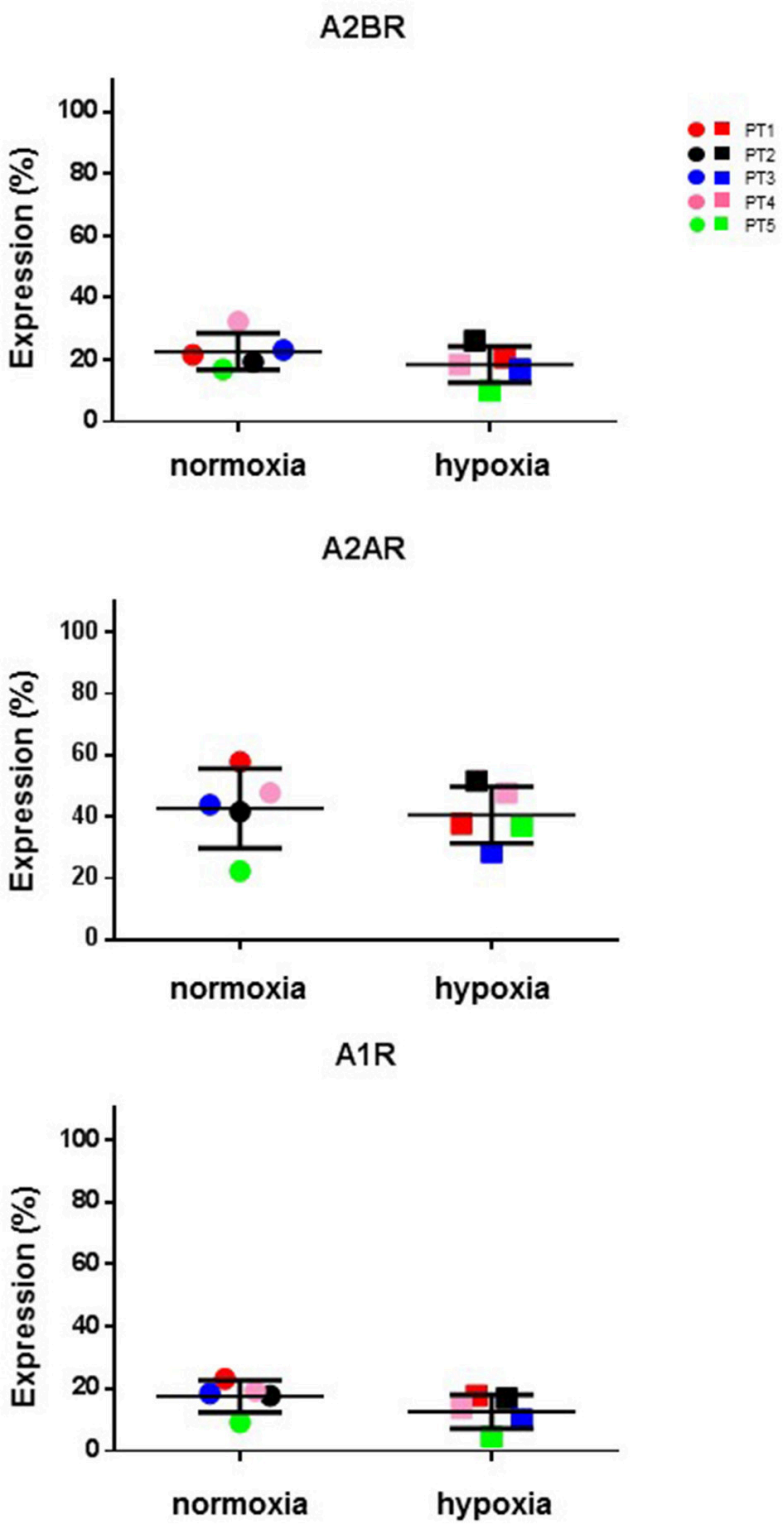
Expression of P1 receptors in normal and hypoxic conditions. Comparative cytofluorometric analysis of A_1_R, A_2A_R and A_2B_R expression in CIK cells stained with primary antibodies to various P1 receptors and detected with fluorescein (FITC)-conjugated secondary antibodies as reported in Materials and Methods. To compare data obtained in hypoxia and normoxia we used unpaired nonparametric test, two-tailed Mann-Whitney for GraphPad Prism 6. Circles, normoxia; squares, hypoxia. Patients are as following: PT1, red; PT2, black; PT3, blue; PT4, pink; PT5, green. Reported data are expressed as mean values ± SD.

Hypoxic microenvironment has been shown to be one of the main drivers for the accumulation of ADO in different cancers and in some cases it increases the expression of CD39 and CD73 (Sitkovsky et al., 2014; Allard et al., 2016). CIK cells have a mixed T- and NK cell-like phenotype and ADO has been shown to hamper anti-tumor functions of these cells. For these reasons, the expression of CD39, CD38, CD203a and CD73 was monitored during CIK differentiation of cells from 5 patients, both in normoxic and hypoxic culture conditions. Figure 6 shows that no significant (*p* > 0.05) differences were evidenced between values obtained in normoxia and hypoxia. ADO receptors are known to suppress immune responses against tumors. Expression analysis of A_1_R, A_2A_R, and A_2B_R was also performed, showing that ADO receptors only underwent minor, non-significant (*p* > 0.05) changes as a consequence of hypoxia (Figure 7).

## Discussion

Adoptive immunotherapy consists in potentiating the anticancer effects of selected immune populations. CIK cells represent a fundamental advancement in immunotherapy because of their ability to eradicate transformed cells, while sparing normal tissues (Vesely et al., 2011; Gajewski et al., 2013). However, in some cases tumors evade the killing activities of these cells. Numerous attempts are being made to enhance the potency and specificity of CIK cells (Wong et al., 2013).

Purine nucleoside adenosine (ADO) is attributed important roles in physiology and pathology. In immunity, ADO has immunosuppressive functions through activation of P1 purinergic receptors expressed by immune cells. ADO is produced from the dismantling of mono- and dinucleotides (ATP and NAD^+^) and their byproducts (ADP, ADPR, AMP) by a set of ectoenzymes (CD39, CD38, CD203a, and CD73), of interest to both basic and clinical research because of their involvement in tumor biology and immune response.

The canonical pathway of ADO production originates from CD39, which metabolizes ATP. However, it was recently demonstrated that CD38 leads to an alternative pathway, whose substrate is NAD^+^ (Horenstein et al., 2013). Both pathways converge into AMP, produced either from ATP/ADP (by means of CD39) or from ADPR (by means of CD203a/PC-1). AMP is then converted to ADO by CD73, the bottleneck enzyme for both adenosinergic pathways. These ectoenzymes are expressed by different normal cells (such as immune effectors) and by tumor cells. In the latter instance, these ectoenzymes grant immunosuppressive properties to the tumor cells by means of ligation of adenosine receptors (A_1_R, A_2A_R, A_2B_R, and A_3_R).

The aim of this study was to investigate expression and function of plasma membrane molecules involved in the adenosinergic pathways in PBMC and CIK cells. Here we showed that CIK cells are characterized by a combination of functional CD38, CD39, CD203a/PC-1, and CD73 ectonucleotidases. Consequently, these cells are potentially able to exploit *ex vivo* the coexistence of the canonical (CD39/CD73) and the alternative (CD38/CD203a/CD73 or CD203a/CD73) pathways. As a result they are able to generate the immunosuppressive purine nucleoside ADO, which either arises from the degradation of ATP or NAD^+^ substrates. Accordingly, the substrates (ATP, NAD^+^) were added to cell cultures, and their products (ADPR, NIC, AMP, ADO and INO) were quantified in the supernatants by means of a dedicated HPLC assay.

At the head of the alternative network converting extracellular NAD^+^ is CD38, a molecule with multiple functions. As an ectoenzyme, CD38 acts as a primary regulator of extracellular NAD^+^ levels (Malavasi et al., 2008). The next component in the ectonucleotidase cascade is CD203a, an ectoenzyme initially known as Plasma Cell-1 (PC-1) (Goding et al., 1998). The interactions between extracellular NAD^+^ and the CD38/CD203a enzymatic tandem can be exploited by CIK to generate AMP. Indeed, the addition of NAD^+^ to CIK cells causes production of ADPR and NIC (products of the enzymatic activity of CD38) and AMP, in the culture supernatants. These results indicate that the outer plasma membrane of CIK cells is equipped with molecules endowed with hydrolytic activities that determine the fate of extracellular NAD^+^. Lastly, we demonstrate that ADPR or AMP added exogenously to CIK cell cultures are further metabolized to produce ADO.

**Figure 8:**
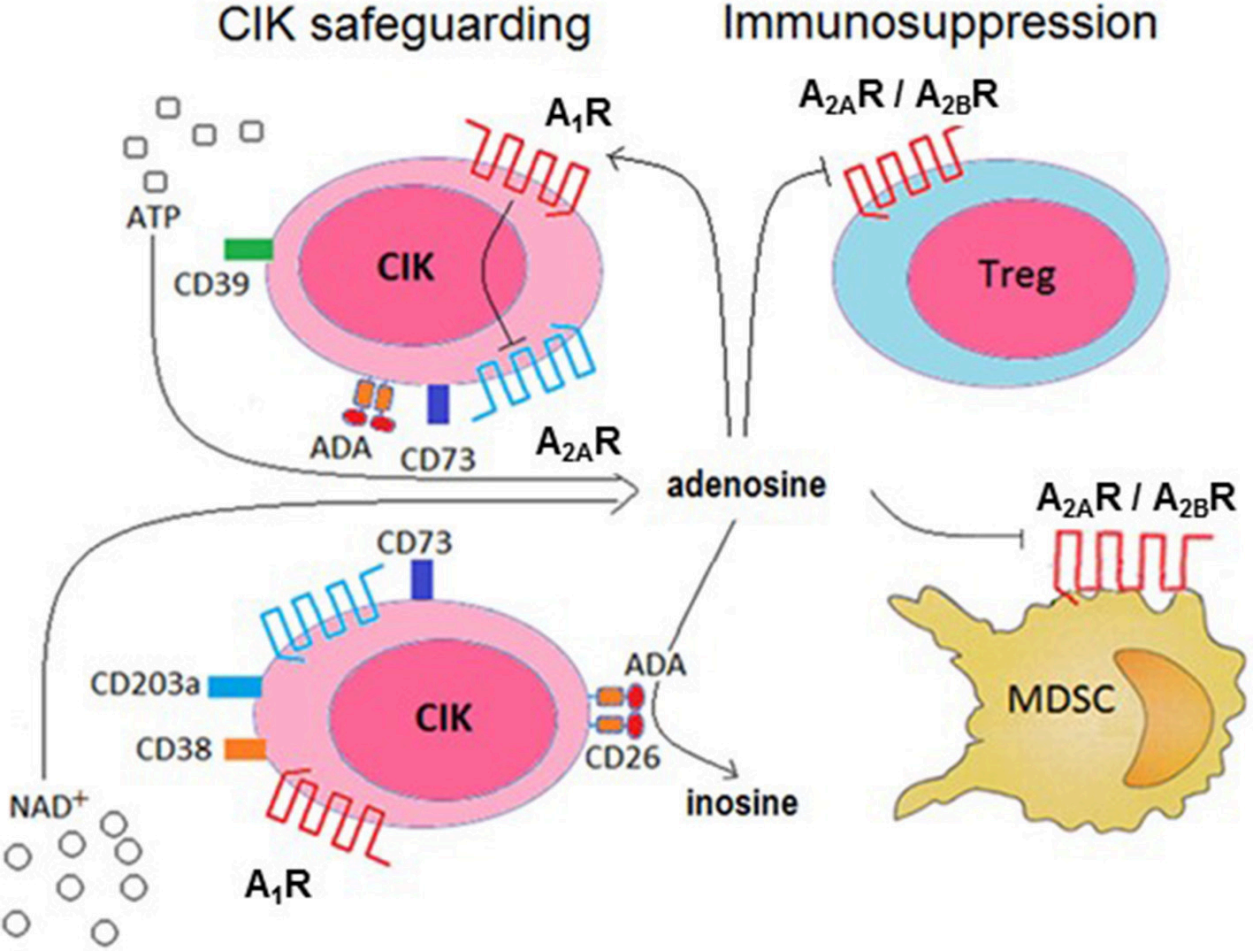
Conceptual model of the role of ADO production, metabolism and receptor ligation in the cytotoxic activity of CIK cells. CIK cells prevent the induction of an autocrine immunosuppression by abrogating A_2A_R-mediated signaling (by A_1_R activation) and deamination of ADO catalyzed by the CD26/ADA complex. In turn, ADO produced by CIK cells can reduce pro-cancer immune responses through paracrine cell inhibition (e.g., Treg, MDSC functions).

Extracellular ADO homeostasis is influenced by the presence of ADA, which irreversibly deaminates ADO, converting it to the related nucleoside inosine. This means that generated ADO is *in vivo* partially transformed by the cells of the surrounding environment, consequently locking the immunosuppressive effects of the nucleoside. Indeed, the low production of ADO in the absence of EHNA (a CD26/ADA inhibitor) is not ascribable to the high expression of ectonucleotidases by CIK cells. There are at least two possible explanations for this lowered production: either up-regulation of the ADA (as inferred by proxy from the increase in its surrogate, CD26) or low expression of CD203a, both of which are observed in differentiated CIK cells. Under such conditions, CIK cells would protect their ability to generate toxic effects against neoplastic cells. The remnant ADO generated by CIK cells in the extracellular medium is however available for binding to P1 purinergic receptors. Alternatively, it can be internalized by nucleoside transporters. This internalization step through the nucleoside transport was not observed in the system analyzed. Indeed, the addition of dipyridamole, an inhibitor of nucleoside transporters, was not followed by an increase in ADO. Things become even more complex considering the finding that CIK cells express high affinity A_1_R and A_2A_R. The presence of the two receptors would support the hypothesis that CIK cells take advantage of the P1 receptors to achieve autocrine signaling and thus self-regulation. Indeed, it is known that many cells express more than one purinergic receptor along with nucleotides degrading ectoenzymes, establishing a regulatory membrane network (Volonté et al., 2006). Therefore, trace amount of pericellular ADO may be sufficient to bind and activate (high affinity) A_2A_R on the surface of CIK cells (inducing a reduction in their immune activity). Nonetheless, A_2A_R activation is reported as being self-inhibited through the action of A_1_R. This effect allow us to postulate that ADO binding to A_1_R expressed by CIK cells can induce an autocrine inhibition of the nucleoside immunosuppressive effect, as depicted in Figure 8. Extracellular nucleoside concentrations can potentially favor (or suppress) the local immune responses, depending on its concentration as well as the relative abundance of P1 receptor subtypes expressed by CIK cells. These P1 receptor subtypes are coupled to different combinations of G-protein family members, namely A_1_ receptors to G_i_/G_o_, A_2a_ receptors to G_s_/G_olf_, and A_2b_ receptors to G_s_/G_q_. Accordingly, engagement of A_2a_ and A_2b_ activates adenyl cyclase, leading to elevated levels of cellular cyclic AMP (cAMP). Instead, A_1_ stimulation inhibits adenyl cyclase, resulting in decreased cellular levels of cAMP. Accordingly, the observed biphasic effect of extracellular ADO could be explained by the peculiar functionality of A_1_ and A_2a_ receptors (Cunha, 2005; Milne and Palmer, 2011).

It has been reported that hypoxia inhibit the proliferation, cytotoxicity and migration of CIK cells *in vitro* (Shi et al., 2013), hampering the effectiveness of CIK therapy. Moreover, the *ex vivo* infiltration of CIK cells in the hypoxic area is hindered, suggesting that a high expression of the complex CD26/ADA might be a condition *sine-qua-non* to warrant cytotoxicity to infused CIK cells. On this premise, we elaborate a model for CD26/ADA action at the CIK cell surface, where human ADA fine-tune ADO concentrations protecting CIK cells from an ADO mediated inhibition of proliferation. Consequently, this versatile strategy would provide a safety lock of CIK cell cytotoxic activities (Figure 8).

Treg cells and mesenchymal derived stromal cells (MDSC) are widely considered immune mediators of peripheral tolerance, playing a pivotal role in limiting anti-tumor immunity (Quarona et al., 2015; Chillemi et al., 2017). Therefore, pathways leading to ADO synthesis on CIK cells may contribute to the induction of paracrine suppression of the Tregs and MDSC immune activities (Figure 8).

A *bona fide* conclusion of the present study is that CIK cells are equipped with a surface machinery leading to ADO production. Moreover, CIK cells are characterized by the expression of ADO receptors with high affinity (A_1_R and A_2A_R): the activation occurs when ADO reaches 1-3 nM concentrations (Burnstock, 2007). CIK cells may produce microenvironmental ADO concentrations above the affinity constant (Ka) of adenosinergic A_1_ and A_2A_ receptors present on the same cells and on immune effectors (e.g., Treg, MDSC). This sequence of events may occur *in situ* in the microenvironment or at a distance. The final outcome is an implementation by CIK cells of an autocrine/paracrine network required for optimal physiological activities. Support for this hypothesis comes from a recent study that showed T helper (Th) 17 cells (CD26^+^) are also equipped with an adenosinergic machinery, which induces a negative regulation of the immune response of Tregs through the production of ADO (Bailey et al., 2014).

In conclusion, the results of this work consented us to highlight critical functional features of human CIK cells, not appreciated yet. Schematically, CIK cells express (i) a panel of extracellular enzymes involved in nucleotide/nucleoside and NAD^+^ metabolism i.e., CD39, CD38, CD203a, and CD73. (ii) The same cells exploit the chain CD26/ADA/ADO to govern the extracellular concentration of ADO. The same effects are likely to be extended at distance, providing negative signals to T lymphocyte populations (De Meester et al., 1999). The results of this work pave the way to verify this hypothesis during the discrete steps of the induction of the CIK cells. Future studies are required to explore the functional implications of our findings within the challenging setting of solid tumors.

## Author contributions

AH, FM, and DF: Conceived and designed the experiments; AH, AC, and RZ: Performed the experiments; AH, AC, FM, VQ, NB, and DF: Analyzed the data; FM, RM, and RG: Contributed reagents; AH, FM, and DF: Wrote the paper.

### Conflict of interest statement

The authors declare that the research was conducted in the absence of any commercial or financial relationships that could be construed as a potential conflict of interest.

## Abbreviations

ADO: adenosine
ADA: adenosine deaminase
AR: adenosine receptor
ADPR: ADP-ribose
CD38: ADP-ribosyl cyclase/cyclic ADPR-hydrolase
CD39: ecto-nucleoside triphosphate diphosphohydrolase (NTPDase)
CD73: ecto-5′-nucleotidase (5′-NT)
CD203a/PC-1: ectonucleotide pyrophosphatase/phosphodiesterase-1 (NPP-1)
CIK: cytokine-induced killer
EHNA: erytro-9 (2-hydroxy-3-nonyl) adenine
GIST: Gastrointestinal Stromal Tumor
PBMC: peripheral blood mononuclear cells
OS: osteosarcoma.
